# Adrenal bleeding due to pheochromocytoma - A call for algorithm

**DOI:** 10.3389/fendo.2022.908967

**Published:** 2022-08-05

**Authors:** Ewelina Rzepka, Joanna Kokoszka, Anna Grochowska, Magdalena Ulatowska-Białas, Martyna Lech, Marta Opalińska, Elwira Przybylik-Mazurek, Aleksandra Gilis-Januszewska, Alicja Hubalewska-Dydejczyk

**Affiliations:** ^1^ Chair and Department of Endocrinology, Jagiellonian University Medical College, Cracow, Poland; ^2^ Department of Endocrinology, Oncological Endocrinology and Nuclear Medicine, University Hospital, Cracow, Poland; ^3^ Department of Radiology, University Hospital, Cracow, Poland; ^4^ Department of Pathomorphology, Jagiellonian University Medical College, Cracow, Poland; ^5^ Nuclear Medicine Unit, Department of Endocrinology, Oncological Endocrinology and Nuclear Medicine, University Hospital, Cracow, Poland

**Keywords:** pheochromocytoma, hemorrhage, adrenal, bleeding, diagnosis, treatment

## Abstract

**Background:**

Adrenal hemorrhage is a rare, usually life-threating complication. The most common neoplasm resulting in spontaneous adrenal bleeding is pheochromocytoma and it accounts for nearly 50% of cases. Currently, the recommendations for the diagnosis and management of patients with adrenal bleeding due to pheochromocytoma are unavailable.

**Materials and methods:**

We performed a database search for all pheochromocytoma patients, diagnosed and treated from 2005 to 2021 in tertiary endocrinology center. 206 patients were identified, 183 with complete data were included in the analysis. We investigated clinicopathological characteristics, treatment and outcomes of hemorrhagic pheochromocytoma cases and characterize our approach to perioperative diagnosis and medical management. Finally our experiences and data from previously published articles concerning adrenal hemorrhage were analyzed to propose a diagnostic and therapeutic algorithm for hemorrhagic pheochromocytomas.

**Results:**

In the whole group, seven patients (4 men and 3 women) with adrenal bleeding were found, (3.8%). Median patient’s age was 49 years (range: 36-78 years). The most common manifestation of adrenal bleeding was acute abdominal pain (5/7). Two patients developed shock. Hormonal assessment was performed in five patients, based on 24-hour urinary fractionated metanephrines with urinary 3-methoxytyramine. Normetanephrine was elevated in all patients, metanephrine and 3-methoxytyramine - in four cases (4/5). Most patients (6/7) had symptoms suggesting pheochromocytoma before hemorrhage – most commonly paroxysmal hypertension (4/7). One patient died, before the diagnosis of adrenal bleeding was made. Diagnostic imaging performed in six out of seven patients revealed adrenal tumor, with median largest diameter equal to 7.4 cm (range: 5-11 cm). Five patients had elective surgery, in one case an urgent surgery was performed. In all cases the diagnosis of pheochromocytoma was confirmed in postoperative histopathology or in autopsy. The perioperative survival rate was 85.7%.

**Conclusions:**

Diagnosis of pheochromocytoma should be always considered in patients with adrenal bleeding, especially with accompanying abdominal pain, hemodynamic shock and previous history of pheochromocytoma-associated symptoms. Lack of proper diagnosis of pheochromocytoma before surgery is associated with an additional perioperative risk. To improve the decision making in this life-threatening clinical situation, based on our results and literature data, we proposed a diagnostic and treatment algorithm.

## Introduction

Spontaneous adrenal hemorrhage is a rare, potentially life-threatening condition. According to literature, the most common neoplasm resulting in spontaneous adrenal bleeding is pheochromocytoma, which accounts for nearly 50% of cases (1). The mortality rate of a ruptured pheochromocytoma is reported to be about 30% (2, 3). Hypovolemia from hemorrhagic shock, heart failure as a result of catecholamine excess, postoperative hypotension or pulmonary oedema are leading causes of perioperative mortality (2, 4). Although most cases of hemorrhage within a pheochromocytoma are spontaneous, sometimes episodes of bleeding could be precipitated by preceding anticoagulant therapy (5), trauma (6) or thrombolysis (7). Lately, the first case of hemorrhage in a pheochromocytoma in the course of SARS-CoV-2 infection was described (8). Moreover, adrenal hematoma could sometimes mimic neoplasm of the adrenal gland (1, 9, 10).

Most publications dedicated to a hemorrhagic pheochromocytoma are case reports, with only several series published to date, including four literature reviews and one case series (1–3, 11, 12).

Therefore we reviewed the records of patients with hemorrhagic pheochromocytoma treated in our department. Our objective was to investigate clinicopathological characteristics, treatment and outcomes of hemorrhagic pheochromocytoma cases and characterize our approach to perioperative diagnosis and medical management. Based on our results and literature data we proposed a diagnostic and treatment algorithm.

## Materials and methods

We performed a database search for pheochromocytoma patients, diagnosed and treated in tertiary endocrinology unit from 2005 to 2021. 206 consecutive patients with pheochromocytoma were identified. Subsequently, 23 cases were excluded due to incomplete medical data (histopathological or imaging) necessary to rule out potential adrenal bleeding. Of the remaining 183 patients with histologically confirmed pheochromocytoma, seven cases with adrenal bleeding confirmed in histopathological examination were found (3.8% of cases). Clinical manifestation, imaging, hormonal status and histopathological results were analyzed. All imaging records of patients suspected for adrenal bleeding were reassessed by one radiologist, skilled in adrenal gland pathology (AG).

24-hour urinary fractionated metanephrines and 3-metoxytyramine were measured using high performance liquid chromatography with electrochemical detection.

Due to the need of urgent hormonal assessment in most cases, only 3 patients have followed the proper dietary restrictions regarding catecholamine-rich products withdrawal before and during 24-hour urine collection.

The summary of patient’s concomitant medications and diet are summarized in **Table 1**.

**Table 1:** Summary of clinical and pathological characteristics of the patients.

	7	6	5	4	3	2	1	Number of the patient
Median value: 49 yrs	64	49	36	48	36	68	78	Age (yrs)
M:W=4:3	W	W	W	M	M	M	M	Sex
Most common – abdominal pain 5/7 (71.4%)	Abdominal pain	Chest pain, flank pain, paroxysmal hypertension, takotsubo cardiomyopathy	Flank pain, chest pain, tachycardia, takotsubo cardiomyopathy	Abdominal pain, flank pain, headache, nausea and vomiting, paroxysmal hypertension	Abdominal pain	Abdominal pain, nausea and vomiting, paroxysmal hypertension followed by hypotension, seizures with short-lasting apnoea, tachycardia	Abdominal pain, nausea and vomiting, hypotension, MOF***	Symptoms of bleeding
Yes -2/7 (28.6%)	No	No	No	No	No	Yes	Yes	State of shock?
Yes– 3/7 (42.9%)	Yes	No	Yes	No	No	Yes	No	Anemia?
Yes - 3/7 (42.9%)	No	No	No	Yes	No	Yes	Yes	Hyper-glycemia?
No – 5/7 (71.4%)	No	No	No	No	No	No	Yes – aspirin and clopidogrel	Platelet-inhibiting medication or anti-coagulants?
Yes – 6/7 (85.7%)	Yes (persistent hypertension)	Yes (paroxysmal hypertension, pallor, anxiety, tachycardia, headache,diaphoresis)	Yes (paroxysmal hypertension).	Yes (paroxysmal hypertension).	Yes (headaches, paroxysmal hypertension, tremor, pallor, tachycardia, anxiety)	Yes (tachycardia, dyspnoea on exertion).	No	Symptoms suggestive of pheochromocytoma before hemorrhage?
Median value 5 months	48 months	3 months	1 month	24 months	5 months	0,5 month	6 months	Time between onset of symptoms and diagnosis of pheochromocytoma (months)
Yes- 6/7 (85.7%)	Yes– at the time of adrenal hemorrhage	Yes – Sixteen months before the hemorrhage	Yes– at the time of adrenal hemorrhage	Yes– at the time of adrenal hemorrhage	Yes – two months before the hemorrhage	Yes – at the time of adrenal hemorrhage	No	Pheochromocytoma suspected preoperatively?
Right – 6/7(85.7%)	Right	Right	Right	Right	Right	Right	Left	Affected side
Intra-tumoral – 6/7 (85.7%)	Intra-tumoral	Intra-tumoral	Intra-tumoral	Intra-tumoral	Intra-tumoral	Intra-peritoneal and Retro- peritoneal	Intra-tumoral	Location of bleeding
Median value 7.4cm	6	7.5	9.5	7.4	6.2	11	5#	Maximum diameter of the tumour (cm)
Median value 1929.9ug/24h	785.8 (2.3 fold)	348.6 (1.02 fold)	9738.8 (28.6 fold) – ongoing hemorrhage 355.6 (1.0 fold) – 3 days later	8759.4 (25.7 fold)	1929.9 (5.7 fold)	N/A	N/A	MN^ (ug/ 24h) Range: 0-341 ug/24h
Median value 4679.9ug/24h	844.4 (1.9 fold)	4679.9 (10.6 fold)	29927.7 (68 fold) – ongoing hemorrhage 1312.6 (3.0 fold) – 3 days later	6034.9 (13.7 fold)	3647.7 (8.3 fold)	N/A	N/A	NMN^^ (ug/ 24h) Range: 0-440 ug/24h
Median value 505.1ug/24h	30.58	404.7	1190.6 – ongoing hemorrhage 539.1 (2.5 fold) – 3 days later	1764.9	505.1	N/A	N/A	3-MT^^^ (ug/ 24h) Range: 0-220 ug/24h
	Yes, 14 days	No	No	Yes, 3 days	Yes, 14 days	Hormonal assessment not done	Hormonal assessment not done	Catecholamine -rich food restrictions before assessment?
	Perindopril Indapamide Carvedilol Omeprazole Iron supplements	Acetylsalicylic acid Amlodipine Atorvastatin Pantoprazole	Enoxaparin Pantoprazole Iron supplements Acetylsalicylic acid Furosemide	Human insulin Enoxaparin Perindopril Atorvastatin Metoprolol	No medications	Hormonal assessment not done	Hormonal assessment not done	Concomitant medications used at the moment of hormonal assessment
Yes 6/7 (85.7%)	Yes 2 miligrams Of doxazosin	Yes 70 miligram Of phenoxybenzamine	Yes 60 miligrams Of phenoxybenzamine	Yes 40 miligrams Of phenoxybenzamine	Yes 12 miligrams Of doxazosin	Yes 6 miligrams Of doxazosin	No	Alpha blockers administration
Elective – 5/7 (71.4%)	Elective	Elective	Elective	Elective	Elective	Urgent	No surgery	Type of surgery
Red blood cell transfusion – 2/6 (30%)	Administration of norepinephrineduring surgery	Uneventful	Red blood cell transfusion	Uneventful	Administration of urapidil during surgery	Red blood cell transfusion	No surgery	Perioperative course
PASS < 4 - 3/5 (60%)	5	4	N/A	1	3	2	N/A	PASS score
Ki-67 <3% 3/4 (75%)	<1%	2%	N/A	N/A	<1%	3,4%	N/A	Ki-67
No – 6/7 (85.7%)	No	No	No	No	No	No	Yes	Death?
Median value 68.5 months	77 months	89 months	60 months	156 months	36 months	11 months	Patient died before the diagnosis	Follow-up period

*M: Man **W: Woman *** Multiorgan failure # tumour size on autopsy.

^MN – metanephrine ^^ NMN – normetanephrine ^^^3-MT – 3-methoxytyramine.

N/A – not available.

### Statistical analysis

Demographic and clinical characteristics were analyzed by the use of frequency tables for categorical variables and by calculation of the median and range for continuous variables. Length of follow-up was presented as range and median value in months, from the time of curative surgery until the last follow-up appointment. The mean values were calculated with standard deviation. The software Statistica 13 made by StatSoft Polska in 2017 was used.

**Figure 1:**
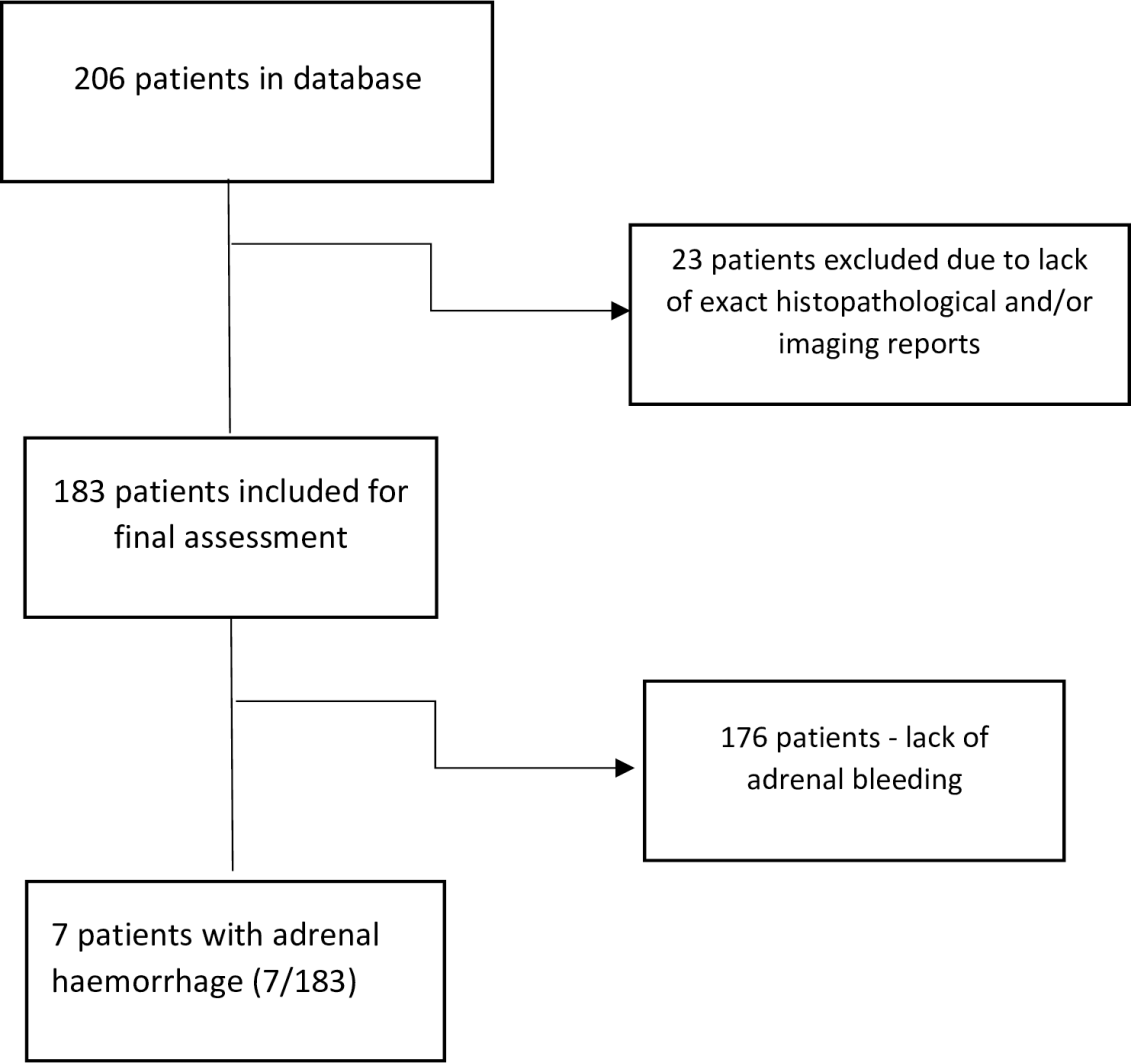
Consort diagram for the final study population.

Consort diagram for the study population is presented on **Figure 1**.

## Results

### Clinical presentation

In the whole cohort, seven patients with adrenal bleeding were found, comprising 3.8% of pheochromocytoma cases (four men and three women). Median patient age was 49 years (range: 36-78 years). All patients had spontaneous adrenal hemorrhage in the absence of recent abdominal trauma. One patient had a history of platelet-inhibiting treatment. The most common manifestation of adrenal bleeding was acute abdominal pain (5 out of 7 patients).

Other symptoms accompanying ongoing hemorrhage were: nausea and vomiting (3/7 patients), paroxysmal hypertension (3/7 patients), flank pain (3/7 patients), chest pain (2/7 patients), tachycardia (2/7 patients), seizures with short-lasting apnoea (1/7 patients), headache (1/7 patients). Two patients developed shock, in one case resulted in multiple organ failure (MOF). In both patients with severe chest pain, takotsubo cardiomyopathy was diagnosed.

Most patients (6/7 cases) had symptoms suggestive of a pheochromocytoma prior to adrenal hemorrhage – most commonly paroxysmal hypertension (4/7). The median time period between onset of symptoms and diagnosis of pheochromocytoma was 5 months (range: 0.5-48 months).

Nevertheless, in four patients diagnosis of pheochromocytoma was made at the time of adrenal hemorrhage, based on severe clinical manifestation, hormonal status and/or imaging. One patient died, before the diagnosis of adrenal bleeding was established. In two patients pheochromocytoma was suspected before the episode of hemorrhage: two months and sixteen months, respectively.

### Imaging

Six out of seven patients had diagnostic imaging: All patients underwent contrast – enhancement computed tomography (CT), with one angio-CT scan. One patient had additional magnetic resonance imaging (MRI) done. Median largest diameter of the lesions was 7.4 cm (range: 5-11 cm). The images revealed different stages and severity of bleeding. The most common features on CT scans were solid-cystic appearance of the lesions, with strong enhancement of the solid component (four patients) and forms of thick-walled hemorrhagic cysts (four patients).

**Figure 2:**
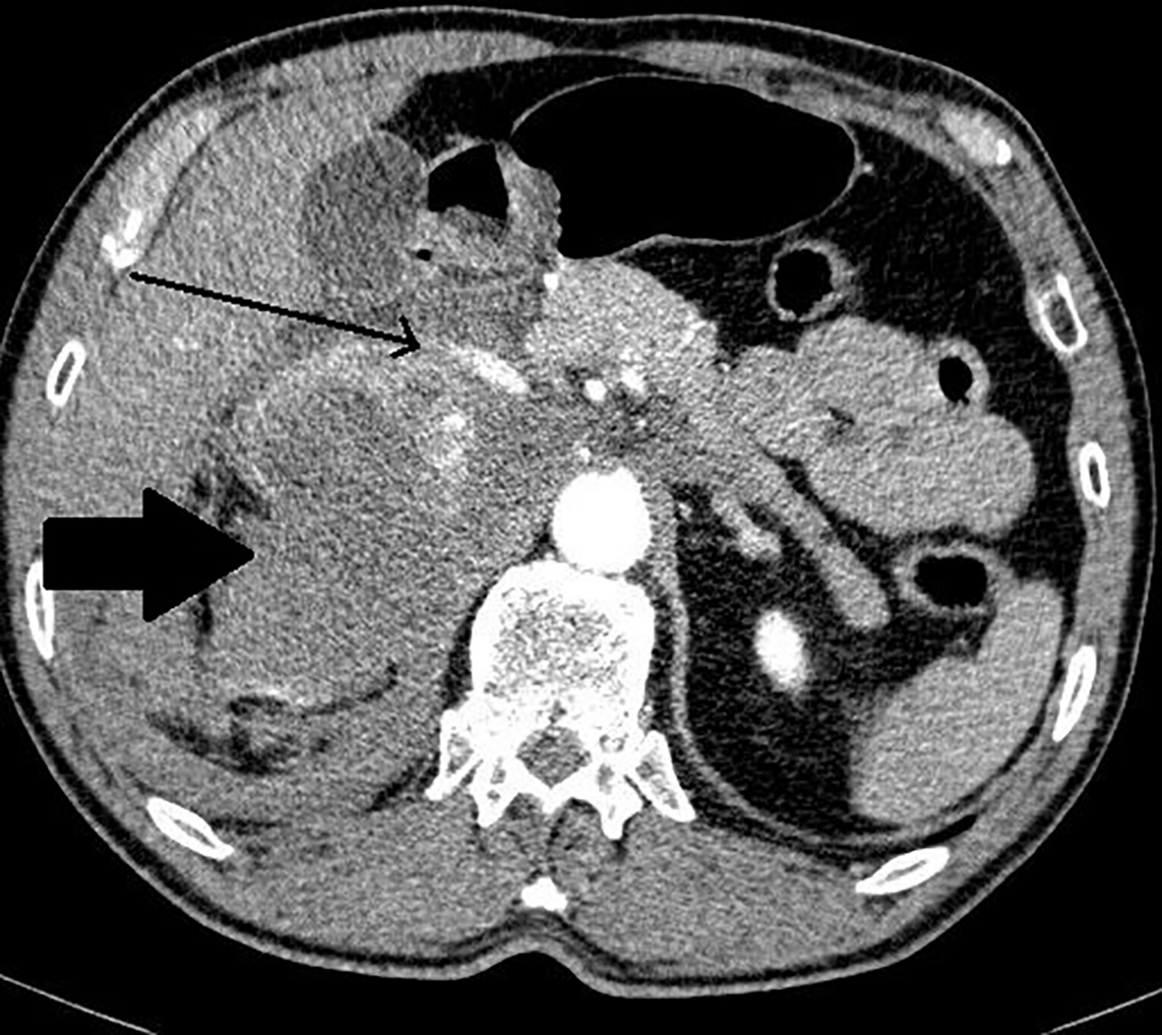
Patient number 2: angio-CT, arterial phase, axial image- right adrenal mass with heterogenous enhancement (thick arrow), suspicion of rupture of central part of the tumour with contrast extravasation (thin arrow). Right adrenal gland is not separately visualized. Left adrenal gland visible, with physiological contrast enhancement.

**Figure 3:**
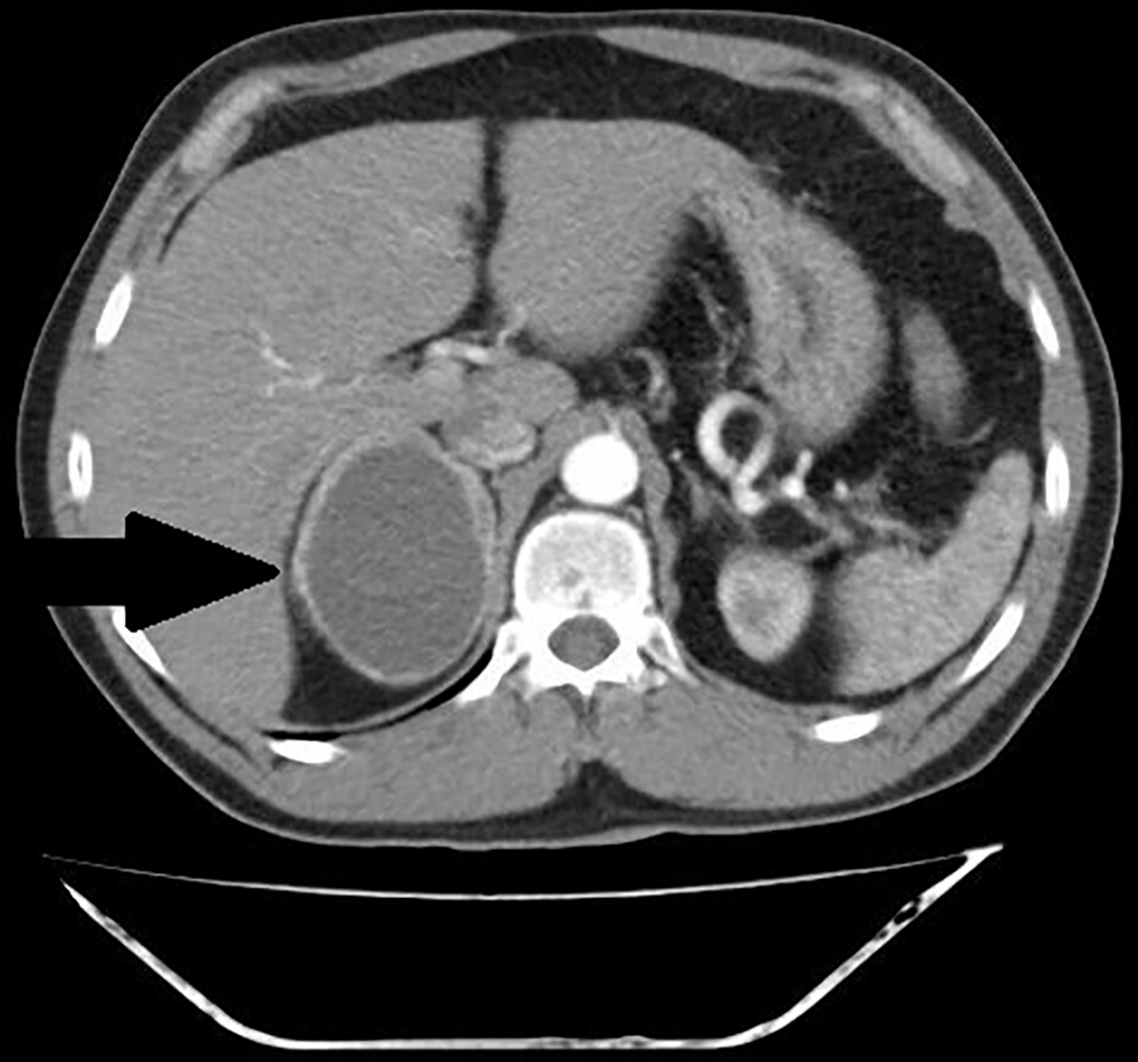
Patient number 4: CT of the abdomen, arterial phase, axial image- thick – walled hemorrhagic cyst of right adrenal gland with strong capsule-contrast enhancement (thick arrow).

**Figure 4:**
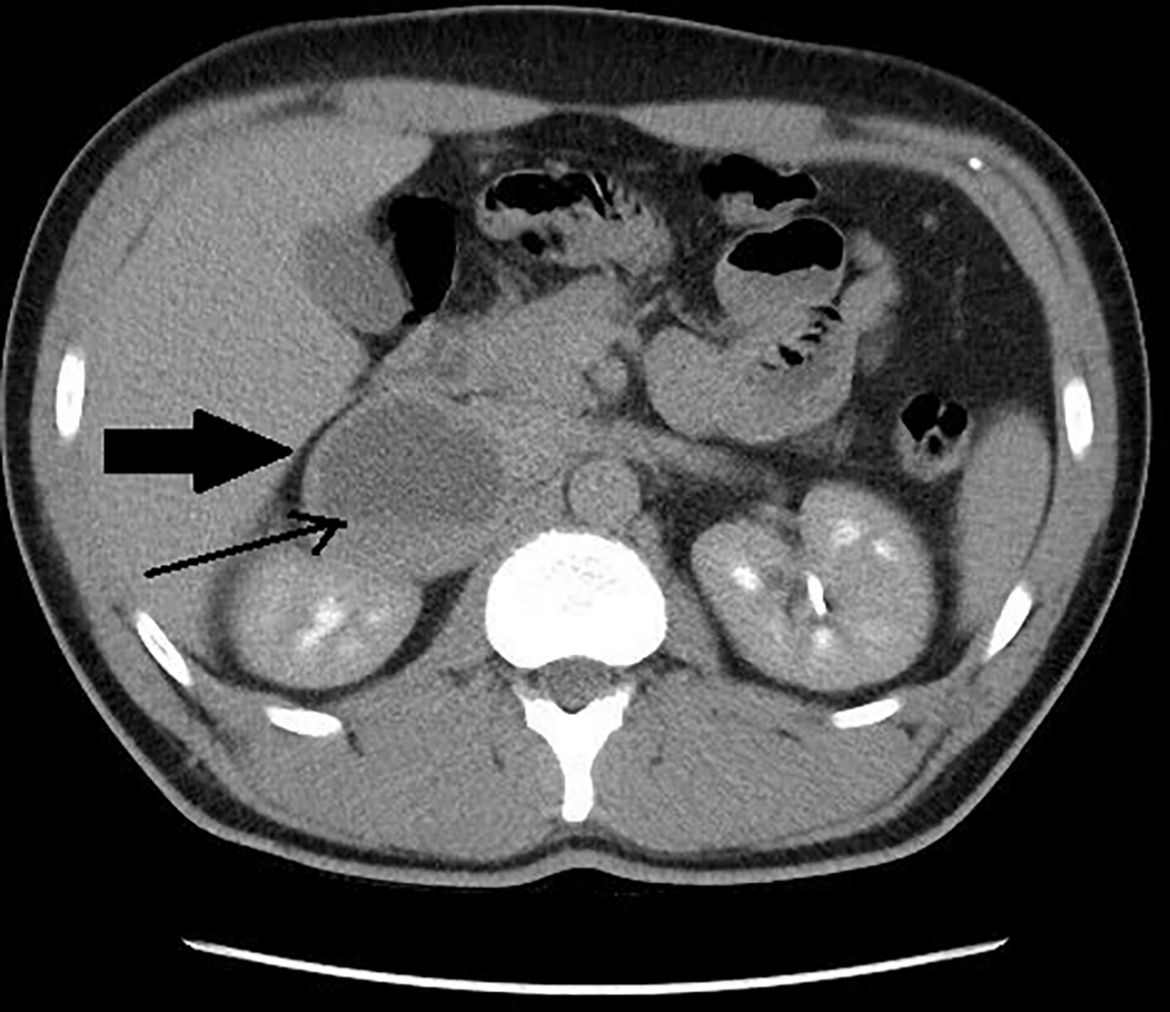
Patient number 7: CT of the abdomen, venous phase, axial image – right adrenal lesion with solid-cystic appearance (thick arrow), central area of fluid attenuation, with fluid-fluid level (thin arrow).

**Figure 5:**
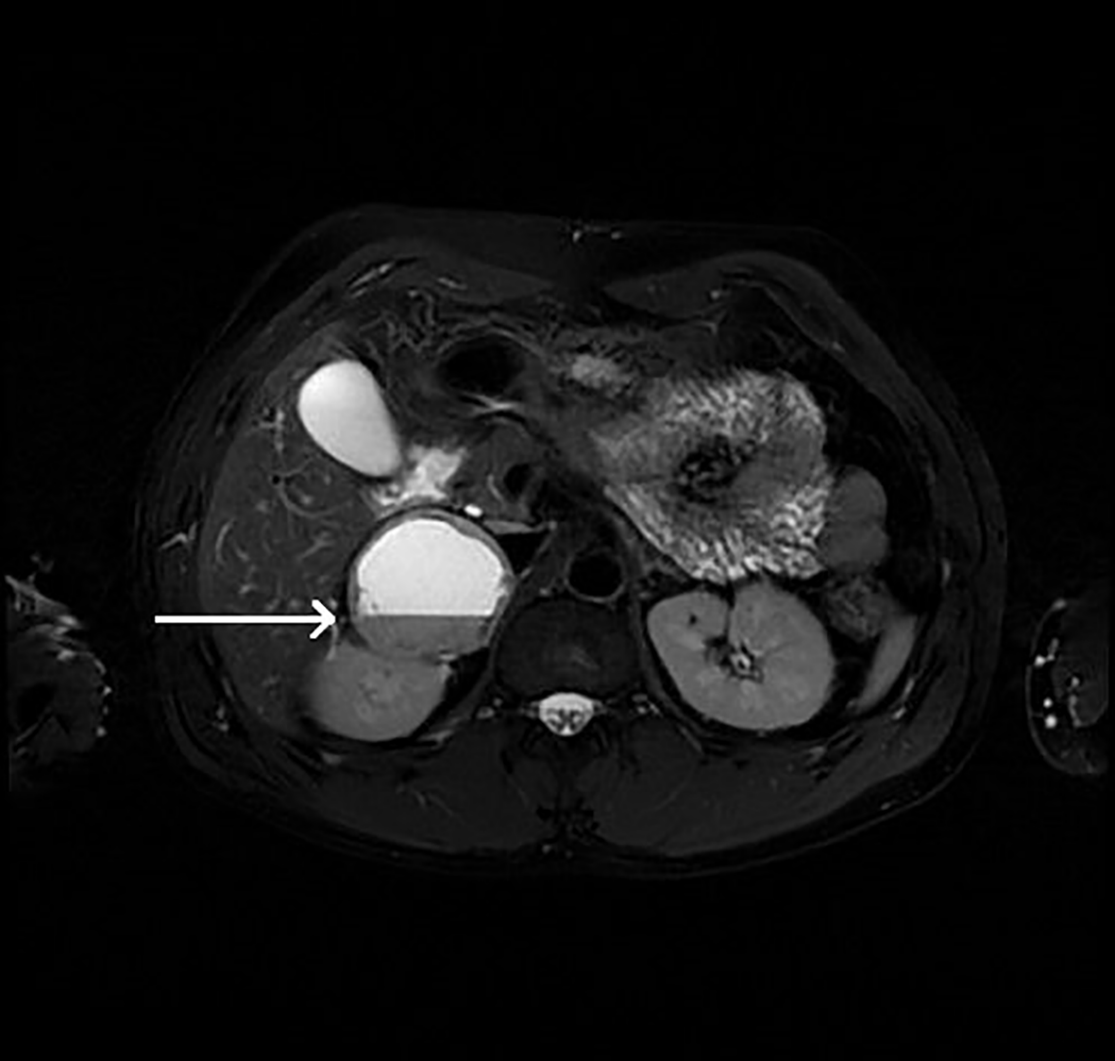
Patient number 5: MRI of the abdomen, T2-weighted axial image – right adrenal lesion with mostly hyperintense signal with fluid-fluid level (thin arrow).

The summary of CT and MRI results are presented in **Table 2**. The CT and MRI images are shown on **Figures 2**–**5**.

**Table 2:** Summary of the results of Computed Tomography (CT) and Magnetic Resonance Imaging (MRI) in six out of seven patients.

Patient’s Number	Computed Tomography	Magnetic Resonance Imaging
1	Not done	Not done
2	Angio-CT: - Right adrenal mass with heterogenous enhancement. - Strong peripheral enhancement. - Suspicion of rupture of central part of the tumour with contrast extravasation to intraperitoneal and retroperitoneal space. - Tumour size: 110x65x80 millimetres.	Not done
3	- Thick – walled hemorrhagic cyst of right adrenal gland. - Strong capsule contrast enhancement. - Central area of fluid attenuation suggestive of recent hemorrhage. - Tumour size:62x60x50 millimetres.	Not done
4	- Thick – walled hemorrhagic cyst of right adrenal gland. - Strong capsule contrast enhancement. - Tumour size: 68x48x74milimetres.	Not done
5	- Right adrenal lesion with heterogenous density. - No contrast enhancement – suggestive of hematoma. - Tumour size: 80x65x95milimetres.	- Right adrenal oval mass. - Low signal intensity on T1-weighted images. - T1 hyper -intense peripheral area, corresponding to blood. - Heterogenous, mostly hyperintense signal on T2-weighted images with fluid-fluid level. - No typical enhancement after contrast administration. - Tumour size: 84x77x96 millimetres.
6	- Right adrenal mass with solid-cystic appearance. - Strong peripheral enhancement (of the solid component) - Tumour size: 75x70x69 millimetres.	Not done
7	- Right adrenal lesion with solid-cystic appearance. - Central area of fluid attenuation, with fluid-fluid level in the posterior part. - Strong peripheral enhancement (of the solid component). - Tumour size: 62x56x56 millimetres.	Not done
Summary	Solid-cystic appearance with strong enhancement of the solid component:4/6 (66.7%) Thick-walled hemorrhagic cyst with strong enhancement of the solid component:3/6 (50%) Thick-walled hemorrhagic cyst with no contrast enhancement: 1/6 (16.7%) Fluid-fluid level:2/6 (33.3%) Contrast extravasation to retro/intraperitoneal space: 1/6 (16.7%) Heterogenous density 2/6 (33.3%)	

### Laboratory assessment

Hormonal assessment concerning pheochromocytoma was performed in five patients, based on 24-hour urinary fractionated metanephrines with urinary 3-methoxytyramine.Normetanephrine was elevated in all patients (from 1.9 to 68-fold above the upper limit). Metanephrine concentration was substantially increased in 4 cases (from 2.3 to 28.6-fold above the upper limit) - in one case it only slightly exceeded the normal range. In all patients, at least one metabolite of catecholamines – metanephrine or normetanephrine - was significantly elevated (more than twice the upper reference limit). Both metanephrines were increased in 4 out of 5 patients subjected to hormonal assessment. 3-metoxythyramine was elevated in 4 patients. The median levels of metanephrine, normetanephrine and 3-methoxytyramine were 1929.9 ug/24h (range: 348.6-9738.8), 4679.7 ug/24h (range: 844.4-29927.7) and 505.1 ug/24h (range: 30.58-1764.9), respectively. Two patients had no hormonal tests – one died before the diagnosis of adrenal hemorrhage was made, in the other, an urgent surgery was not preceded by evaluation of hormonal activity. However, the radiological images suggested pheochromocytoma and typical pharmacological treatment was implemented. Anemia was observed in three out of seven cases, similar to hyperglycemia, which was noted also in 3/7 patients.

### Treatment

In six out of seven patients pheochromocytoma was suspected preoperatively, based on clinical manifestation, hormonal status or imaging. One patient had been admitted to hospital in hemorrhagic shock and multiple organ failure and had died before an etiology of adrenal bleeding was revealed. The diagnosis of hemorrhagic pheochromocytoma was made on autopsy. In five cases, the elective surgery was performed, preceded by a two- week pharmacological treatment with alpha-receptor blockers. In one case, due to patient’s prolonged hemodynamic instability despite supportive care, accompanied by rapid decrease of hemoglobin level and radiological suspicion of intraperitoneal hemorrhage, four-day alpha-receptor blockage was administered, followed by the urgent surgery. None of the patients has transcatheter arterial embolization (TAE) procedure performed before the surgery. In perioperative care two patients had indications to red blood cell transfusion. In one case administration of urapidil was necessary due to significant hypertension during surgery. One patient required intravenous infusion of norepinephrine intraoperatively.

### Histopathological examination

**Figure 6:**
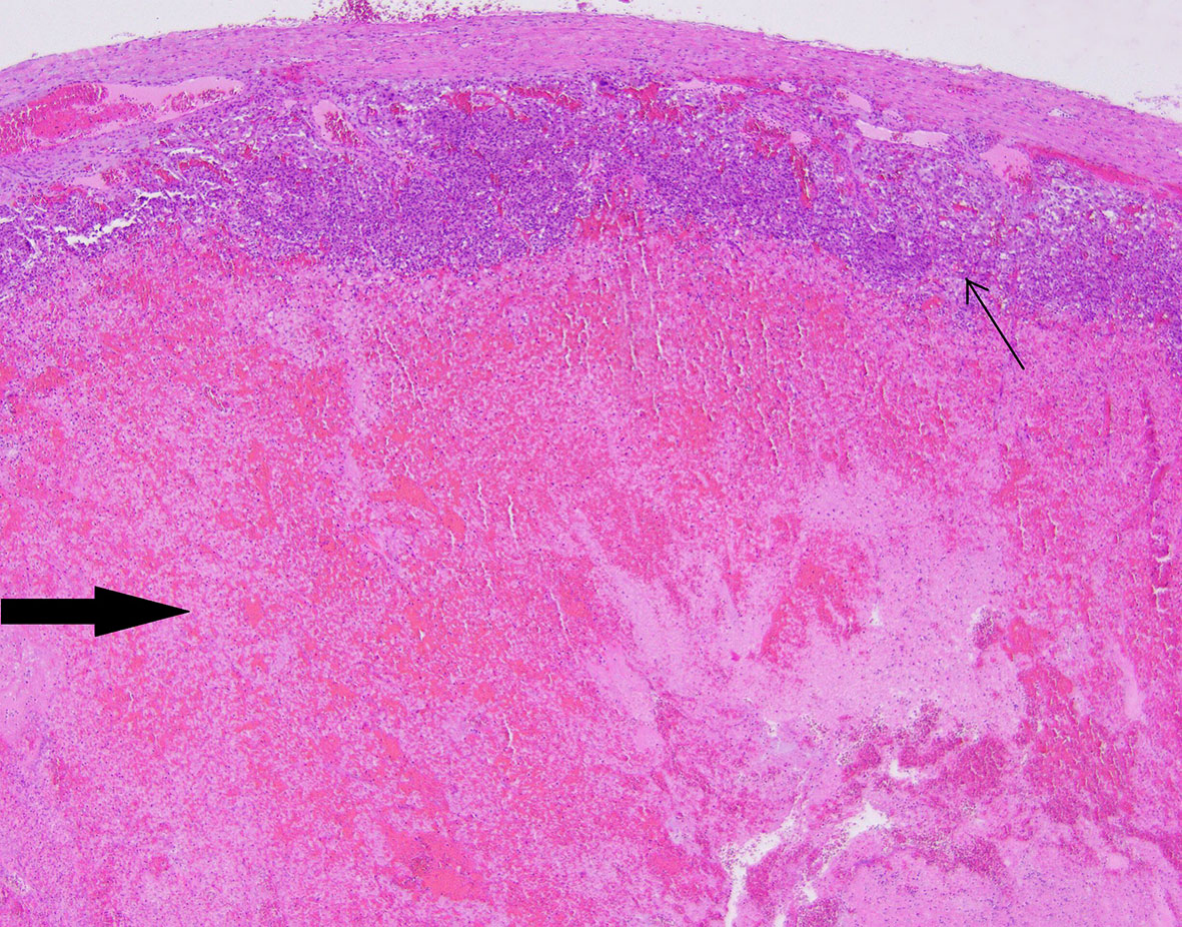
Patient number 4: extensive, diffuse hemorrhage in the central part of the pheochromocytoma (thick arrow), the neoplasm’s tissue is present as subcapsular narrow rim (thin arrow).

**Figure 7:**
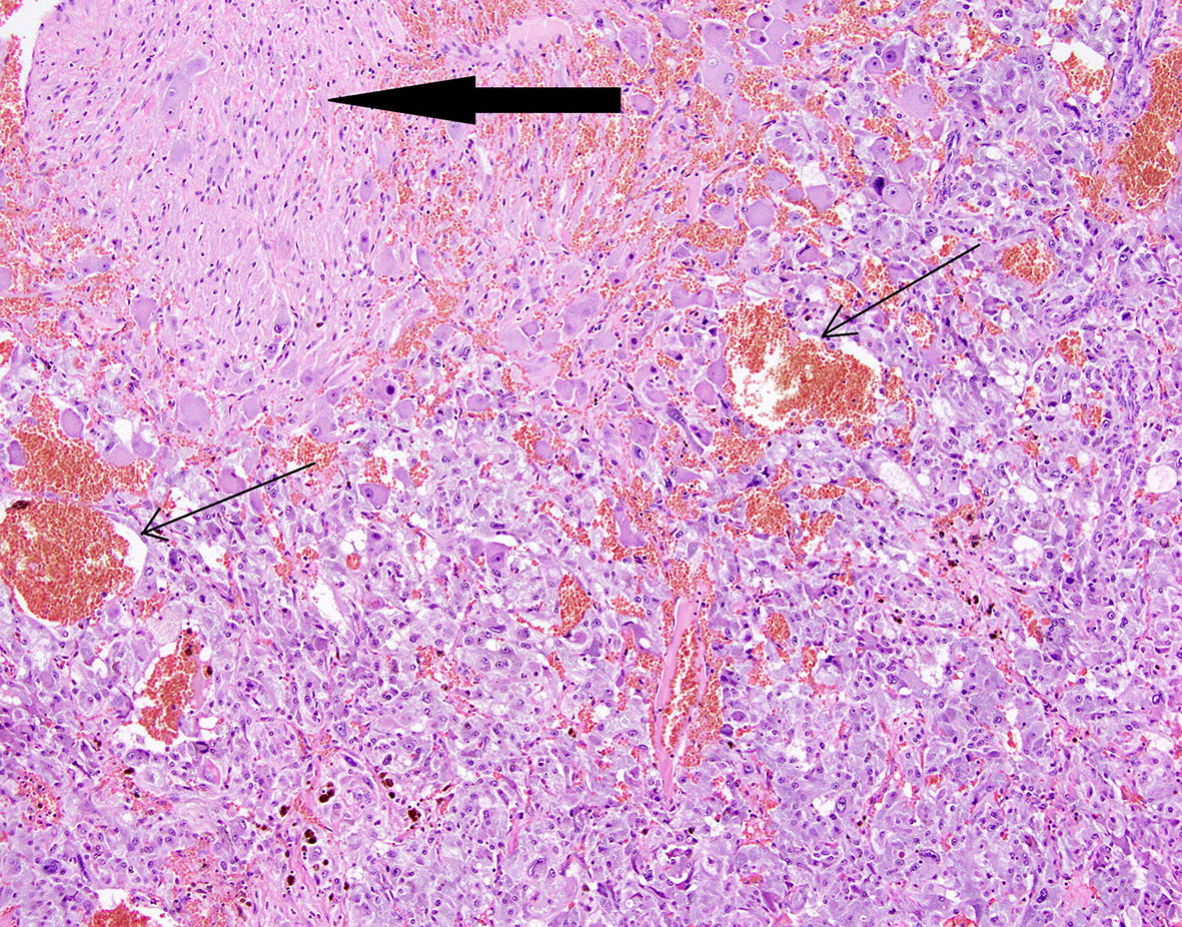
Patient number 3: composite pheochromocytoma and ganglioneuroma (ganglioneuroma component - thick arrow). Hemorrhages are present within pheochromocytoma tissue (thin arrows).

**Figure 8:**
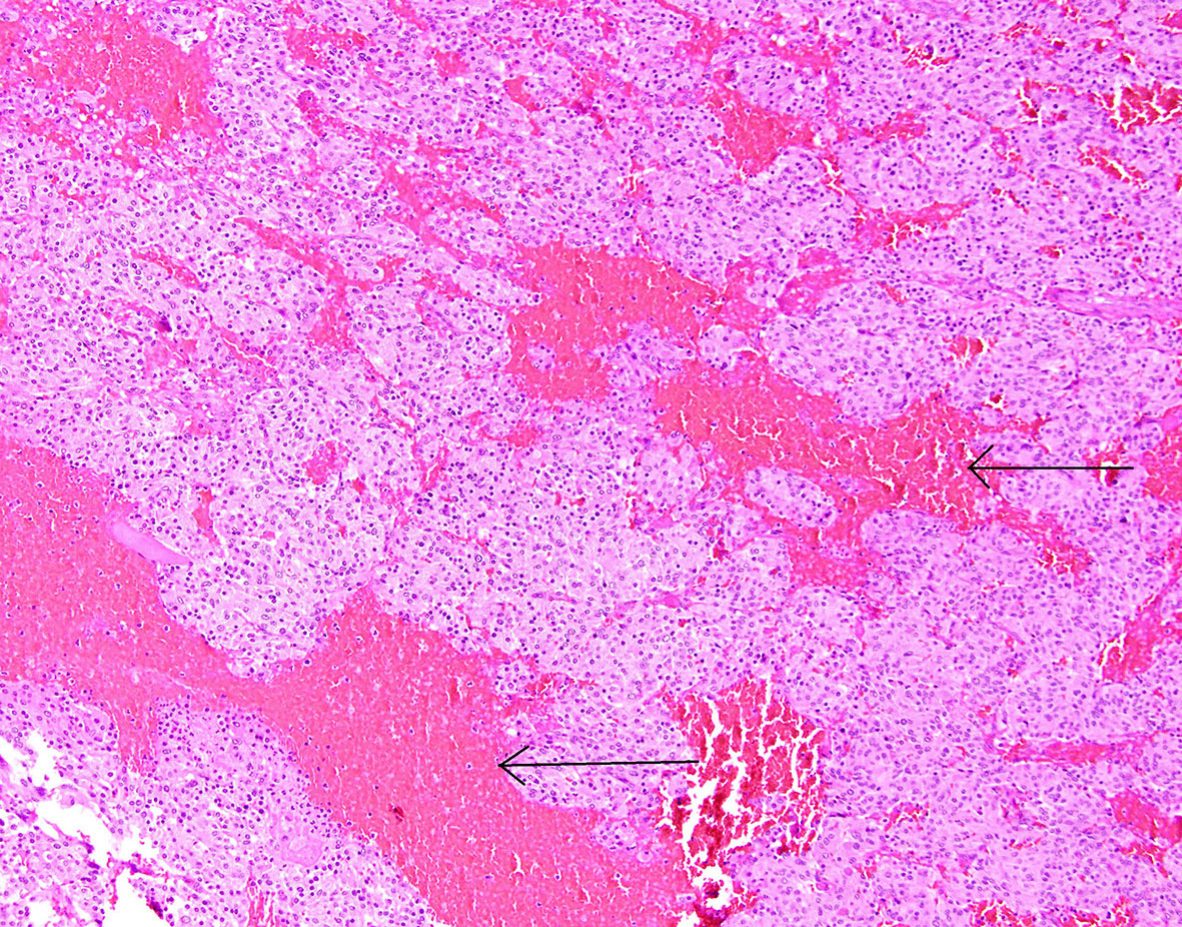
Patient number 2: central part of the pheochromocytoma with irregular, diffuse hemorrhages between tumour nests (thin arrows).

In all cases the diagnosis of pheochromocytoma was histopathologically confirmed by immunohistochemistry - typical positive staining: synaptophysin and chromogranin and the presence of S100+ sustentacular cells in neoplasm. In all patients massive hemorrhagic changes in the tumor’s tissue was confirmed. PASS score was defined precisely in 5 patients. In three cases, it was no higher than 3. In two remaining patients, PASS score was 5 and 4, respectively. Because of massive hemorrhage, in two patients it couldn’t be determined. Histopathological images are shown on **Figures 6**–**8**.

### Outcome and follow – up

In our group, the survival rate was 85.7%. Only one patient died, before the initial diagnosis of adrenal bleeding, shortly after admission to the department. The median follow up period was 68.5 months (Range: 11-156 months). All six patients who survived, remained at good condition, without any evidence of recurrence on last follow-up visit.

The clinicopathological characteristics of the patients are summarized in **Table 1**.

## Discussion

Pheochromocytoma is the most common neoplasm responsible for spontaneous adrenal hemorrhage (1). In our data we found the clinically significant hemorrhage in 3.8% of patients with pheochromocytoma.

According to the literature, the most common symptom of hemorrhagic pheochromocytoma is abdominal pain of acute onset, which is in accordance with our work (2, 3, 11). The pain is presumably connected with local compression of surrounding viscera and adjacent structures by the lesion but it might be also caused by catecholamine over-secretion, accompanied by stimulation of alpha-adrenergic receptors, constriction of intestinal vascular smooth muscle and contraction of the ileocolic sphincter (3, 10, 13).

The status of shock in patients with hemorrhagic pheochromocytoma can be the result of massive bleeding, but also a sudden fall in the blood catecholamine level or excessive release of catecholamines, followed by cardiogenic shock (2, 4, 14). In our group, two out of seven patients developed shock. In one case it was accompanied by massive intra – and retro-peritoneal bleeding and anemia, which suggested a hemorrhagic shock as an important element of patient’s hemodynamic destabilization. In second case, multiorgan failure, hyperglyaemia and acidosis without evident anemia raised suspicion that catecholamine crisis could play a key role in patient’s deterioration.

Catecholamines oversecretion often leads to tachyarrhytmias, of which severe and refractory sinus tachycardia, atrial fibrillation and ventricular tachycardia seem to be the most common (15).

The rare complication of catecholaminergic crisis, which could accompany hemorrhagic pheochromocytoma is Takotsubo cardiomyopathy, characterized by transient left ventricular systolic dysfunction (16, 17). In our group, Takotsubo cardiomyopathy was diagnosed in two patients.

As spontaneous adrenal hemorrhage is an insidious medical condition, it might remain unrecognized with lethal consequences (18, 19). Similar course of the disease was reported in one of our patients, who died few hours after admission, without a diagnostic statement.

On CT and MRI scans, the attenuation value or signal intensity of an adrenal hematoma depends on the stage of the bleeding. On CT, adrenal bleeding presents heterogenous, hyperdense appearance, which decreases over time (20–23). Contrast-enhanced CT may demonstrate active contrast extravasation in the setting of acute bleeding (21). A homogenous density greater than 50 Hounsfield Units (HU) is characteristic for hematoma, especially in cases with acute hemorrhage (22).

On MRI, the acute stage of adrenal bleeding is characterized by isointense or slightly low signal intensity on T1-weighted images and markedly low signal intensity on T2-weighted images, whereas in the early subacute phase hyperintense T1- and hypointense T2- weighted images are observed (20, 21). The high signal intensity appears at the periphery of the hematoma on T1-weighted images about 7 days after onset of the hemorrhage (23). Moreover, in the late subacute stage T2-weighted images become hyperintense (20, 21). In comparison to that, hyperintense signal on T2-weighted images with avid enhancement after contrast administration is characteristic for pheochromocytoma (20, 21, 24).

The radiographic findings similar to hemorrhagic adrenal neoplasm can be observed in patients with adrenal pseudocysts (hematomas), thus making the differential diagnosis notably difficult, especially in terms of a clot in a hemorrhagic cyst mimicking a solid component or a cyst presenting thick and irregular walls. Undoubtedly, it should be taken into consideration in biochemically negative patients (9, 23). Intravenous contrast injection on MRI or CT could help in stating a correct diagnosis since the presence of enhancement in adrenal mass raises the suspicion for an underlying tumor (20, 21, 23, 24). Interval imaging to assess possible resolution of hematoma could be helpful in patients without biochemical evidence of a functional tumor to exclude neoplasm (1). In some cases, sedimentation level could be seen in adrenal lesions as a sign of a previous hemorrhage (25).

In one of our patients, on MRI examination, low-signal intensity with peripheral hyperintense signal on T1-weighted images and heterogenous, mostly hyperintense signal on T2- weighted images were shown. Uncharacteristically, there was no typical contrast enhancement after both iodinated contrast (on CT) and gadolinium contrast (on MRI) administration in this patient. It could be explained by massive hemorrhage filling in and damaging the tumor’s tissue, which was confirmed histopathologically. In this regard, the appearance of the non-contrast enhancing pheochromocytoma in our series was quite different from the cases of hemorrhagic pheochromocytomas reported by the others (20, 21, 23).

All patients with hemorrhagic adrenal mass should undergo hormonal assessment to exclude hormonally active tumors. Both catecholamine and cortisol levels should be evaluated (1, 26).

The preferred tests for biochemical diagnosis of pheochromocytoma is measurement of plasma or urinary free metanephrines with the use of high performance liquid chromatography, especially with mass spectrometry. In patients at low risk for a PPGL, the assessment of plasma free metanephrines has similar diagnostic accuracy as those in urine, however in patients, for whom biochemical proof of PPGL is the objective, the measurement of plasma free metanephrines is recommended. The measurement of urinary fractionated metanephrines might have higher ratio of false-positive results, since their levels are more affected by the diet (27–32). Increments in any plasma metabolite in excess of twice the upper reference limit or increases in two metabolites indicate a high likelihood of pheochromocytoma (27). To optimize the diagnostic performance, use of personalized age-specific reference intervals for plasma normetanephrine and gender-specific reference intervals for urinary metanephrines is recommended (31) . 

Due to physiological stress, levels of catecholamines can be elevated in patients with adrenal hemorrhage, even without pathologically confirmed pheochromocytoma, which may lead to misdiagnosis.

Previous work showed that in cases of adrenal bleeding other than underlying pheochromocytoma, concentrations of urinary fractionated metanephrines only slightly exceeded normal laboratory range (50-100% above the upper limit) in about 30% of cases, with predominant normetanephrine level increase – moreover, the patients did not present symptoms indicative of an excess of catecholamines (33). Kyoda et al. revealed normal or slight elevation to no more than 3-fold the upper limit of catecholamines or their metabolites in the plasma and 24-hour urine in patients with hemorrhagic pseudotumor (9).

We also reviewed all the articles published from 2005 to 2022 in PubMed, dedicated to adrenal bleeding with known results of urinary or plasma catecholamines or metanephrines (8, 10, 12, 14, 16, 17, 24, 34–61). The analysis showed that catecholamines or metanephrines (plasma or urine) were elevated in 38.9% of patients with adrenal bleeding without pheochromocytoma. In most cases only normetanephrine/noradrenaline exceeded the normal range. In one patient slight increase in urinary adrenaline was observed. The urinary or plasma normetanephrine levels were increased to less than 3-fold the normal upper limit (maximum 2.1-fold the upper normal limit for plasma normetanephrine, 2.9-fold for urinary normetanephrine), meanwhile urinary or plasma noradrenaline levels were increased no higher than 1.6 -fold the upper limit of normal range. Contrarily to that, in patients with hemorrhage due to a pheochromocytoma, in most cases both urinary or plasma catecholamines/metanephrines were elevated (21/24, 87.5%). In patients with assessed levels of urinary or plasma metanephrines, at least one metabolite exceeded 2-fold the upper normal limit. Based on that, we might conclude, that adrenal bleeding in non-pheochromocytoma patients results in slightly, non-specific elevation of normetanephrine/noradrenaline to no more than 3-fold the upper limit of normal in about 30-40% of cases. In patients with hemorrhagic pheochromocytomas, most often both catecholamines (or their derivatives) are elevated.

**Figure 9:**
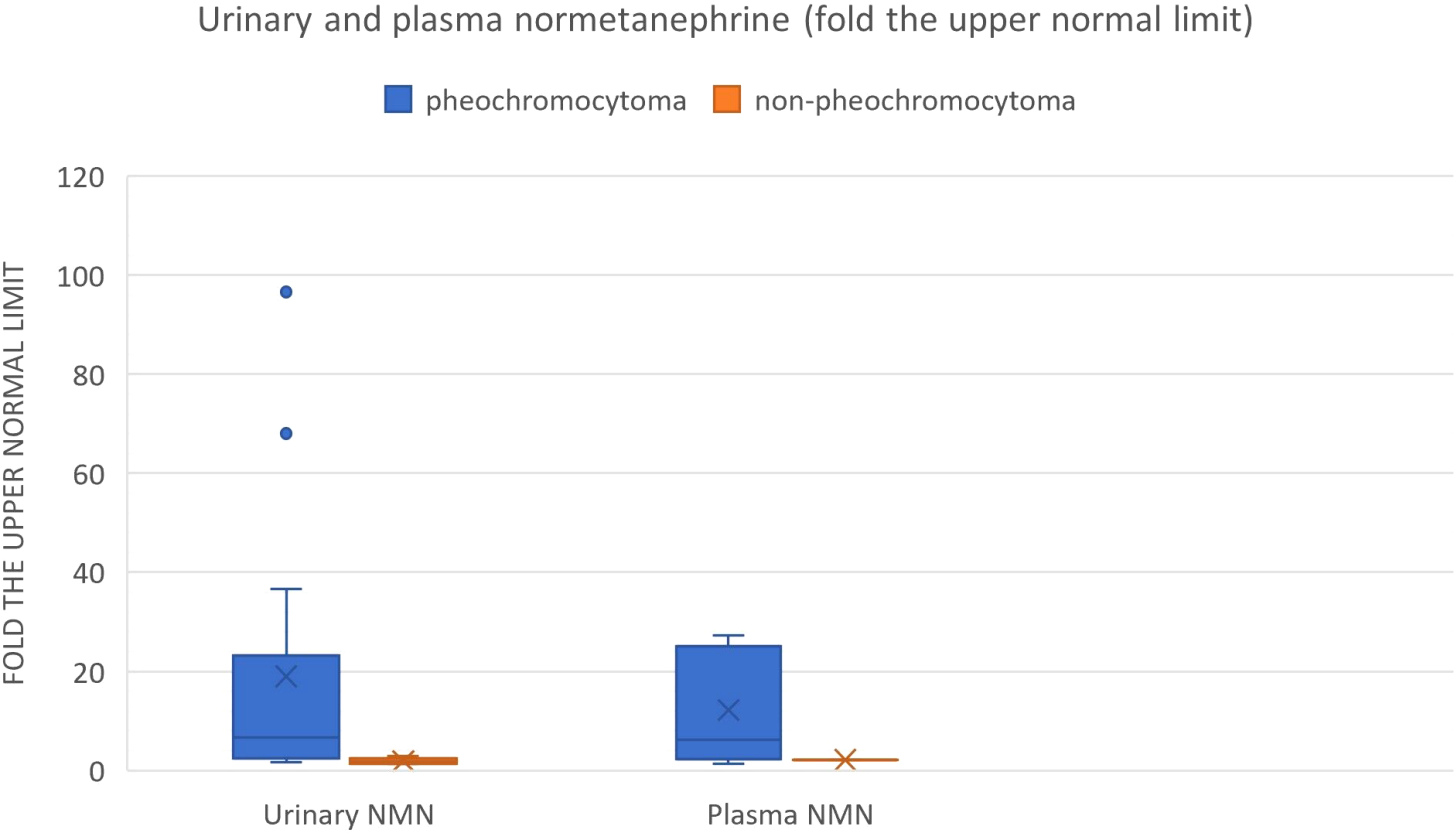
Urinary and plasma normetanephrine levels in patients with hemorrhagic pheochromocytoma and adrenal bleeding without pheochromocytoma based on the literature (3, 10, 12, 14, 20, 25–29, 30–39, 40–49, 50–54.).

**Figure 10:**
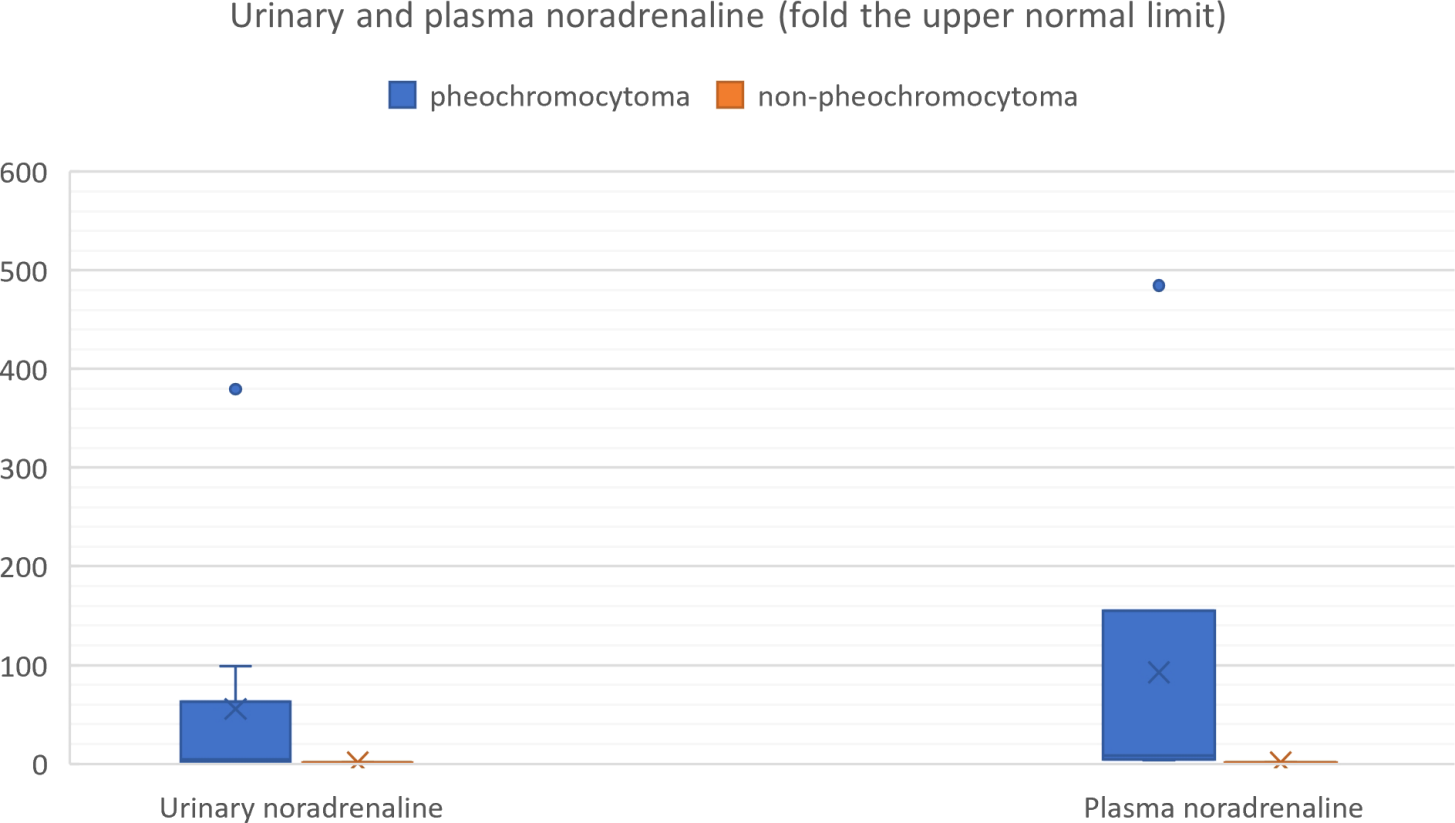
Urinary and plasma noradrenaline levels in patients with hemorrhagic pheochromocytoma and adrenal bleeding without pheochromocytoma based on the literature (3, 10, 12, 14, 20, 25–29, 30–39, 40–49, 50–54).

The results are shown in **Table 3** and on **Figures 9**, **10**.

**Table 3:** Levels of catecholamines/metanephrines in patients with hemorrhagic pheochromocytoma and adrenal bleeding without pheochromocytoma based on the literature (8, 10, 12, 14, 16, 17, 24, 34–61).

	Hemorrhagic pheochromocytoma (our cases included)- No of patients: 24 (8, 12, 14, 16, 17, 24, 34, 35, 37–39, 41–43, 53, 55, 56, 59–61)	Hemorrhagic non-pheochromocytoma:- angiomyolipoma –No of patients: 1 (54)- adrenal pseudocysts – No of patients:17 (10, 36, 40, 44–52, 57–58)
Urinary/plasma metanephrines/catecholamines within normal range	4.2% (1/24)	61.1% (11/18)
**Only** urinary/plasma **normetanephrine/noradrenaline** elevated	8.3% (2/24)	33.3% (6/18)
**Both** urinary/plasma metanephrines/catecholamines elevated	87.5% (21/24)	5.6% (1/18)
Mean urinary normetanephrine elevation (fold the upper normal limit)+/- SD	18.8 ± 26.3 (range: 1.6-68)	1.89 ± 0.6 (range:1.27-2.9)
Mean urinary metanephrine elevation (fold the upper normal limit)+/- SD	15.0 ± 14.1 (range:1.02-47.8)	Not applicable
Mean plasma normetanephrine elevation (fold the upper normal limit)+/- SD	12.16 ± 10.7 (range 1.3-27.3)	2.1
Mean plasma metanephrine elevation (fold the upper normal limit)+/- SD	16.52 ± 15.6 (range: 1.7-28.6)	Not applicable
Mean urinary noradrenaline elevation (fold the upper normal limit)+/- SD	55.61 ± 112.1 (range:1.1-379.58)	1.4 ± 0.2(range 1.1-1.6)
Mean urinary adrenaline elevation (fold the upper normal limit)+/- SD	67.34 ± 69.0 (range:1.7-176.5)	1.2
Mean plasma noradrenaline elevation (fold the upper normal limit)+/- SD	92.65 ± 175.8 (range:3.6-484.4)	1.1 ± 0
Mean plasma adrenaline elevation (fold the upper normal limit)+/- SD	105.64 ± 177.9 (range:5.8-460.0)	Not applicable

Emergency surgery with regard to hemorrhagic pheochromocytoma has a high mortality rate of 40-45% (1, 3, 11, 22). Operation without proper pharmacological treatment jeopardizes patient’s safety due to high risk of catecholamine crisis and postoperative hypotension (4, 56). However, in some groups of patients, i.e. with massive intraperitoneal bleeding, ineffective preoperative management or embolization, emergency exploratory laparotomy becomes the only treatment option (2, 4, 11, 59).

Alpha-adrenergic receptor (AR) blockers are the first-choice medications in preoperative management of pheochromocytoma. Phenoxybenzamine and doxazosin are the most commonly used. The main differences between those two drugs concern selectivity and affinity of drug-receptor interactions. Phenoxybenzamine as the non-selective, non-competitive alpha-adrenergic receptor blocker binds irreversibly with both alpha 1 and alpha 2 – AR, which results in strong, long-acting inhibition. Doxazosin competitively inhibits only alpha 1-AR. For this reason, treatment with phenoxybenzamine gives more effective AR-receptors blockade and better control of hypertension, at the cost of higher risk of postoperative hypotension and other side effects, like reflex tachycardia, oedema and nasal congestion, comparing to doxazosin. Selective alpha-AR antagonist is more likely to be used in combination with additional antihypertensive drugs, i.e. calcium channel blockers (62, 63).

In our work, three patients were treated with phenoxybenzamine, in three patients doxazosin was administered , as phenoxybenzamine was no longer broadly available in Poland. In one patient, who had been prepared with 12 milligrams of doxazosin in a maximum dose, administration of urapidil was needed during surgery due to significant hypertension.

According to literature, transcatheter arterial embolization (TAE) preoperatively, particularly in a case of a ruptured pheochromocytoma, in patients who don’t respond to red blood cell transfusions and initial conservative management, might be preferred initial therapy (1, 3, 7, 11, 53, 59). It can help to stabilize the patient’s state, perform biochemical testing and administer pharmacological treatment before undergoing operation, which improves survival (3, 11, 59). Another possible benefit of trans-arterial embolization preceding elective surgery is consolidation of the hematoma and tumor shrinkage (3, 53). There are only three cases evaluating catecholamine levels around TAE – two of them reported post-TAE peak of circulating catecholamines, resulted in hypertension, nausea, epigastric pain or constipation (11, 64). Therefore, careful observation of patient’s vital signs and symptoms is necessary after TAE procedures. Marti et al. included embolization in proposed treatment algorithm for patients with hemorrhagic adrenal neoplasms (1).

In the literature, there are only several studies comprising cases of pheochromocytoma hemorrhage (1–3, 11, 12). There were summarized, together with our work, in **Table 4**. In comparison to our group, previously reported cases were characterized by higher proportion of emergency surgery (47-58.3% vs 14.3% in our work) and worse survival outcomes (27-41.7% of patients died vs 14.3% in our group). It could be explained by less number of patients with diagnosis of pheochromocytoma stated preoperatively but also more cases of severe, intraperitoneal and retroperitoneal bleeding (21-100% of intraperitoneal bleeding and 50-55% of retroperitoneal bleeding vs both – 14.3% in our work). Kobayashi showed that failed preoperative diagnosis of hemorrhagic pheochromocytoma was an independent factor for poor prognosis. Moreover, there was a strong correlation between correct preoperative diagnosis and elective surgery. Hemodynamic instability had a significant influence on the correct diagnosis of a pheochromocytoma (2). In recent years, improvement of survival rates despite the comparable proportion of more severe hemorrhage can be seen (11). It seems to be connected with better availability of imaging studies, development of new procedures in adrenal bleeding, including TAE and emphasis on preoperative management and preferentially elective type of surgery.

**Table 4:** Clinicopathological characteristics and treatment results of hemorrhagic pheochromocytoma in our study and previously published series.

	Our work	Kobayashi T. et al. *(2)* 2005	Habib M. et al. *(3)* 2010	Marti J.L et al. *(1)* 2011	Hanna J.S et al. [*(12)*] 2011	Edo N. et al. *(11)* 2018
**Number of patients**	7	50	53	64	12	74
**Median age (years)/ (range)**	49 (36-78)	50 (15-80)	50.1 (15-80)	50	51.5(31-76)	50.1 (15-84)
**Gender (men:women)**	4:3	25:25	27:26	35:29	7:5	41:33
**Acute abdominal pain**	5 (71.4%)	40 (80%)	42 (79%)	N/A	10 (83.3%)	58 (78%)
**Shock**	2 (28.6%)	29 (58%)	30 (57%)	N/A	2 (16.7%)	38 (51%)
**Prior history of tumor associated symptoms**	6(85.7%)	21 (42%)	N/A	N/A	N/A	N/A
**suspicion of pheochromocytoma before operation**	6 (85.7%)	20 (40%)	N/A	N/A	N/A	N/A
**Tumor side (right:left:bilateral)**	R’ -8(85.7%) L’’-1 (14.3%) B’’’-0	R-27 (54%) L-22 (44%) B-1 (2%)	R-30 (56%) L-22 (42%) B-1 (2%)	R-32 (50%) L-31 (48%) B-1 (2%)	N/A	N/A
**Median size of the tumour (cm)**	7.4	N/A	N/A	7	N/A	N/A
**Intratumoral hemorrhage**	6 (85.7%)	12 (24%)	13 (25%)	N/A	0	18 (24%)
**Intraperitoneal hemorrhage**	1 (14.3%)	13 (26%)	13 (24%)	N/A	12 (100%)	15 (21%)
**Retroperitoneal hemorrhage**	1 (14.3%)	25 (50%)	27 (51%)	N/A	0	41 (55%)
**Surgery**	6 (85.7%)	41 (82%)	41 (77%)	51 (80%)	9 (75%)	62 (84%)
**Emergency surgery**	1 (14.3%)#	29 (58%)	29 (55%)	N/A	7 (58.3%)	35 (47%)
**Elective surgery**	5 (71.4%)	12 (24%)	12 (23%)	N/A	2 (16.7%)	27 (37%)
**Surgery not performed**	1 (14.3%)	9 (18%)	9 (17%)	9 (14%)	3 (25%)	12 (16%)
TAE*	0	N/A	3 (5%)	4 (6%)	N/A	7 (10%)
**Survived**	6 (85.7%)	33 (66%)	36 (68%)	N/A	7 (58.3%)	54 (73%)
**Died**	1 (14.3%)	17 (34%)	17 (32%)	N/A	5 (41.7%)	20 (27%)

N/A – not available.

‘R – right.

‘’L – left.

‘’’ B – bilateral.

* TAE – trans-arterial embolization.

# including one case with urgent surgery after four-day alpha-receptor blockage.

However, in the article summarizing 12 cases of intraperitoneal hemorrhage due to pheochromocytoma, conservative management was connected with 100% mortality (3/3 patients), contrarily to the survival rate of 71.4% (5/7) in patients treated by emergency surgery. Those results therefore stressed the need for more aggressive and faster surgical interventions in case of massive intraperitoneal hemorrhage in pheochromocytoma patients (12). Successful hemodynamic stabilization by TAE procedures in patients with ruptured pheochromocytomas accompanied by intraperitoneal hemorrhage has been also described (34, 35).

None of the previous series has focused on histopathological results in a hemorrhagic pheochromocytoma. Nevertheless, in the literature, reported cases of adrenal bleeding in pheochromocytomas with high PASS score, metastases and recurrences can be found (53, 55, 59, 64). Moreover, Xin-Gao et al. showed that intratumoral hemorrhage was abundantly detected in histologically high-graded pheochromocytomas (65). In our group, the PASS score was no higher than 3 in three cases, but it exceeded 3 in two remaining patients. Nevertheless, in two patients it couldn’t be determined. Moreover in four out of five patients with known hormonal status, the level of dopamine metabolite, 3-metoxythyramine was substantially elevated. It is known that dopamine-producing tumors are more likely to present aggressive course of the disease and metastasize (66). Therefore, we suggest that in patients with hemorrhagic pheochromocytoma, careful follow-up is of great importance, since due to a massive bleeding, histopathological assessment is especially difficult and carries a risk of a PASS score underestimation and underprediction of other histopathological factors suggesting malignant course of the disease.

The potential risk factors for the pheochromocytoma hemorrhage are difficult to establish, since the most cases are diagnosed at the moment of ongoing adrenal bleeding, which may affect levels of circulating catecholamines and tumor size. The previous cases with repetitive assessment of catecholamines showed that their levels can fluctuate after the episode of adrenal bleeding within pheochromocytoma, probably because of tumor tissue damage and necrosis (60, 67). In one of our patients, concentration of urinary metanephrines decreased significantly three days after the hemorrhage (see **Table 1**, Patient Number 5). Catecholamine levels can be also influenced by pharmacological treatment with vasopressors (17). Some authors suggest, that the mechanism of hemorrhage may be explained by excessive amount of circulating catecholamines leading to vasoconstriction of the draining venules and extensive necrosis of pheochromocytoma, with subsequent decrease of catecholamines secretion, increase in blood flow into the tumor and high intratumoral pressure. It can result in rupture of the thin walled adrenal venules and tumor capsule. Moreover, anatomical characteristics of the adrenal gland vessels may also contribute to an increase in intratumoral pressure because the blood outflow vessels in the adrenal glands are much narrower than inflow vessels and the muscle bundles surrounding the central and external adrenal veins are arranged eccentrically, leading to turbulent flow within the vein (1, 3, 18, 23, 38, 59, 60).

In addition, AR-blockers have been reported as a potential cause of tumor rupture in pheochromocytoma (60).

In the group of 64 patients with hemorrhagic pheochromocytoma, described by Marti et al. median tumor size was 7 cm, which is similar to our observations (7.4 cm in our group). Based on the earlier studies (1–3) and our research, adrenal bleeding seems to be predominantly observed in pheochromocytomas localized in right adrenal glands. In two recent series, it was reported more often in men than women (1, 11), but some works, as well as our observations, did not confirm the predominance of men (2, 3, 12). The median age at diagnosis of hemorrhage in reported series, including ours, was about 50 years (1–3, 11, 12).

The major limitations of our study is its retrospective design. Moreover, a non-surgical character of our department, might result in bias in patient selection with respect to mortality, treatment strategies, and timing of surgery. Some patients with more severe bleeding and hemodynamic instability might have be transferred directly to surgical departments, which could explain low rate of intra- and retro-peritoneal bleeding in our patients and high percentage of preoperatively diagnosed pheochromocytoma. It could also lead to an underestimation of a number of hemorrhagic cases among all pheochromocytoma patients.

**Figure 11:**
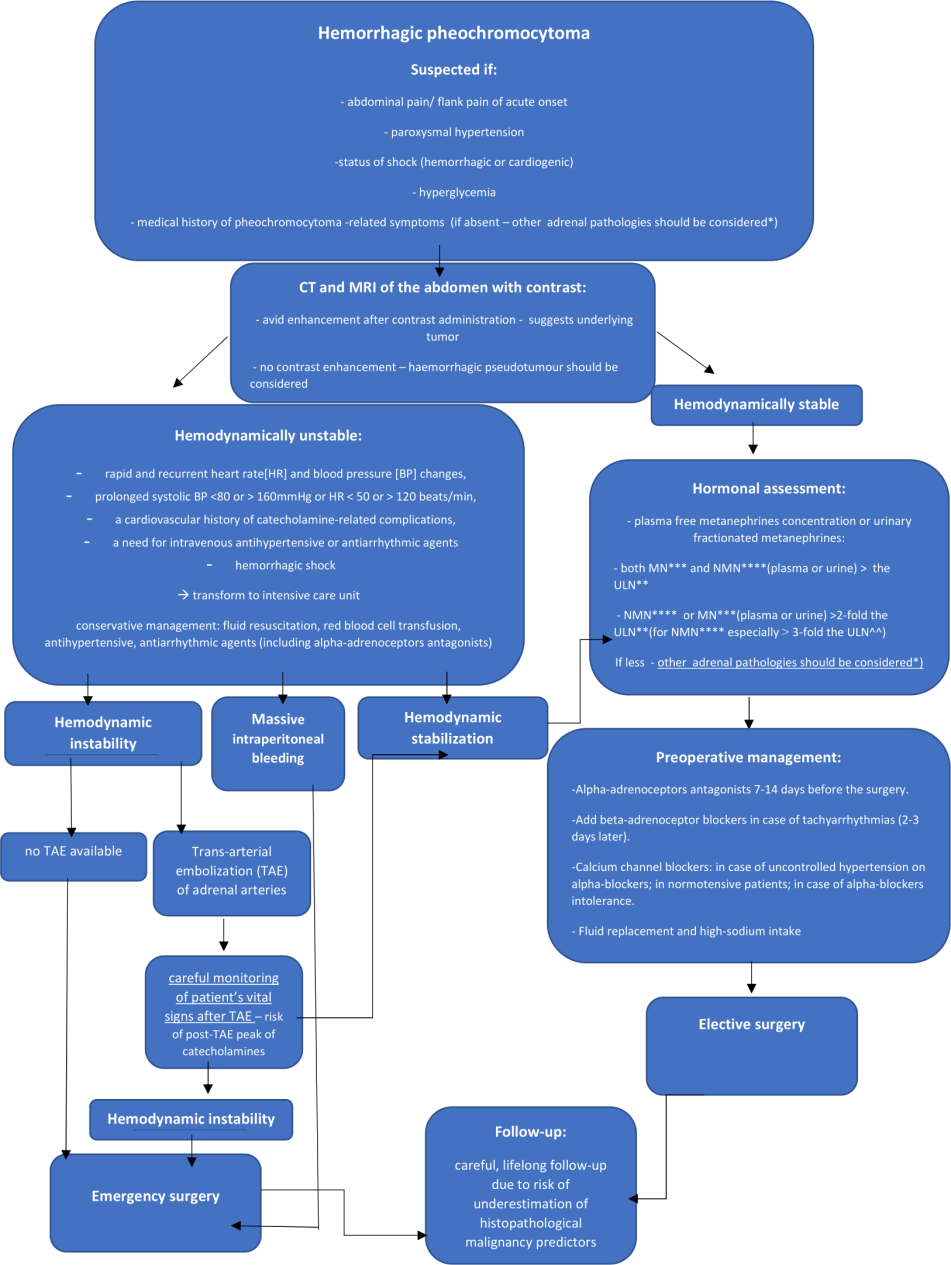
Algorithm for the diagnosis and treatment of adrenal hemorrhage in the course of pheochromocytoma. *mainly hemorrhagic pseudotumour, metastases, myelolipoma, primary adrenocortical carcinoma ** The upper limit of normal *** metanephrine****normetanephrine

Based on the current available literature and our own experience, we suggest an algorithm for the diagnosis and treatment of adrenal hemorrhage in the course of pheochromocytoma (1–4, 7, 11, 12, 14, 15, 22, 23, 26, 27, 35, 38, 40, 43, 62, 68). It is shown on **Figure 11**. We have also summarized the medical management in hemorrhagic pheochromocytoma:

If hemorrhagic pheochromocytoma is suspected, perform urgent CT scan or MRI of the abdomen. It can help to assess the severity of bleeding and reveal underlying adrenal mass. Patients with unstable hemodynamic conditions due to catecholamine-induced arrythmias (rapid and recurrent heart rate [HR] and blood pressure [BP] changes, prolonged systolic BP <80 or > 160mmHg or HR < 50 or > 120 beats/min, a cardiovascular history of catecholamine-related complications, a need for intravenous antihypertensive or antiarrhythmic agents) or hemorrhagic shock should be treated in intensive care units (15). For the latter, fluid resuscitation and red blood cell (RBC) transfusion are the mainstay of treatment. In hemodynamically stable cases, perform hormonal assessment of cortisol and catecholamines. Increments in either metanephrine or normetanephrine in excess of twice the upper reference limit (for normetanephrine especially at least 3-fold) or increases in two metabolites indicate a high likelihood of pheochromocytoma (27). All hemodynamically stable patients should be qualified to elective surgery preceded by pharmacological management. Start the preoperative treatment of pheochromocytoma patients with alpha-adrenoceptors antagonists 7-14 days before the surgery. Beta-adrenoceptor blocking agents can be added in case of tachyarrhytmias, only after adequate pretreatment with alpha-adrenoceptors blockers (not earlier than 2-3 days after alpha-blockade). The β -blockers implementation without preceding α-blockers treatment may result in hypertensive crisis due to inhibition of β2-adrenoceptor mediated vasodilatation with coexistence of unopposed α -adrenoceptor-mediated vasoconstriction (27, 68). Cardioselective β1-adrenoceptors blockers are preferable. Labetalol and Carvedilol, with a higher potency for β- than α-adrenergic receptors should be avoided in monotherapy in pheochromocytoma patients (15, 27, 68). Calcium channel blockers are good option for the patients with uncontrolled blood pressure despite α -adrenoceptor blockers use, in case of α-adrenoceptor blockers intolerance and severe side effects or in patients with normal blood pressure or only intermittent hypertension to avoid α -adrenoceptor induced hypotension. (27, 62, 69). In such cases, ivabradine can also be used (15). In case of hypotension, unrelated to blood loss, consider potential β2-receptor overstimulation and administer a non-selective β2-adrenoceptor blocking agents (propranolol) or phenylephrine. For all patients adequate fluid replacement (with intravenous saline infusion in the evening before operation) and high-sodium intake prior to surgery is also important (62). Suggested optimal presurgical heart rate and blood pressure targets are: seated blood pressure <130/80mmHg and an upright systolic blood pressure > 90mmHg; heart rate 60-70 and 70-80 bpm in seated and upright position, respectively (15). For the patients with hypertensive crisis, intravenous administration of phentolamine sodium, nitroprusside, nicardipine or urapidil is recommended (69). In hemodynamically unstable patients despite conservative management, perform trans-arterial embolization (TAE) of adrenal arteries, followed by careful monitoring of patient’s vital signs. Patients unsuccessfully treated by TAE procedures, hemodynamically unstable despite conservative management, in case of unavailable TAE or with massive intraperitoneal bleeding should be qualified to emergency surgery. After the operation of hemorrhagic pheochromocytoma, careful life-long follow-up of the patients seems to be the most appropriate.

## Conclusions

Adrenal bleeding is a rare, life-threatening complication of pheochromocytoma, which constitutes a diagnostic and therapeutic challenge. Proper diagnosis is essential for adequate preparation for surgery. Clinical symptoms including abdominal pain, accompanying by hemodynamic shock and previous history of pheochromocytoma-associated symptoms should alert about potential risk of adrenal bleeding. The emergency surgery in patients with adrenal bleeding from ruptured pheochromocytoma can be connected with significantly higher morbidity and mortality. Stabilizing arterial embolization could be an effective initial therapy in patients with hemodynamic instability, giving the opportunity for better preoperative management. In patients with hemorrhagic pheochromocytoma, careful postoperative follow-up is of great importance, since due to a massive bleeding, histopathological assessment may be less credible.

## Data availability statement

The original contributions presented in the study are included in the article/supplementary material. Further inquiries can be directed to the corresponding author.

## Ethics statement

The study was approved by Jagiellonian University Ethical Committee (approval number:1072.6120.218.2020). Written informed consent for participation was not required for this study in accordance with the national legislation and the institutional requirements.

## Author contributions

ER, data collection, data analysis, manuscript drafting, manuscript editing and approval, study coordination. JK, data analysis, manuscript editing and approval. AG and MU-B, data collection, analysis, manuscript editing, and approval. ML, data collection, manuscript editing, and approval. MO, manuscript drafting, editing, and approval. EP-M and AH-D, manuscript editing and approval, study coordination. AG-J, data collection, data analysis, manuscript drafting, editing and approval, and study coordination. All authors contributed to the article and approved the submitted version.

## Funding

The study is sponsored as the part of the Special Purpose Grant for Young Scientists by the Jagiellonian University, Medical College, Cracow, Poland, SAP N41/DBS/000117).

## Conflict of interest

The authors declare that the research was conducted in the absence of any commercial or financial relationships that could be construed as a potential conflict of interest.

## Publisher’s note

All claims expressed in this article are solely those of the authors and do not necessarily represent those of their affiliated organizations, or those of the publisher, the editors and the reviewers. Any product that may be evaluated in this article, or claim that may be made by its manufacturer, is not guaranteed or endorsed by the publisher.
